# Microbial Niche Diversification in the Galápagos Archipelago and Its Response to El Niño

**DOI:** 10.3389/fmicb.2020.575194

**Published:** 2020-10-23

**Authors:** Scott M. Gifford, Liang Zhao, Brooke Stemple, Kimberly DeLong, Patricia M. Medeiros, Harvey Seim, Adrian Marchetti

**Affiliations:** ^1^Department of Marine Sciences, University of North Carolina at Chapel Hill, Chapel Hill, NC, United States; ^2^Department of Civil & Environmental Engineering & Earth Sciences, University of Notre Dame, Notre Dame, IN, United States; ^3^Department of Ocean Sciences, University of California, Santa Cruz, Santa Cruz, CA, United States; ^4^Department of Marine Sciences, University of Georgia, Athens, GA, United States

**Keywords:** marine, metagenomics, ecology, biogeochemistry, DOC, El Niño, bacteria, Galápagos Archipelago

## Abstract

The Galápagos Archipelago is located at the intersection of several major oceanographic features that produce diverse environmental conditions around the islands, and thus has the potential to serve as a natural laboratory for discerning the underlying environmental factors that structure marine microbial communities. Here we used quantitative metagenomics to characterize microbial communities in relation to archipelago marine habitats, and how those populations shift due to substantial environmental changes brought on by El Niño. Environmental conditions such as temperature, salinity, inorganic dissolved nutrients, and dissolved organic carbon (DOC) concentrations varied throughout the archipelago, revealing a diversity of potential microbial niches arising from upwelling, oligotrophic to eutrophic gradients, physical isolation, and potential island mass effects. The volumetric abundances of microbial community members shifted with these environmental changes and revealed several taxonomic indicators of different water masses. This included a transition from a *Synechococcus* dominated system in the west to an even mix of *Synechococcus* and *Prochlorococcus* in the east, mirroring the archipelago’s mesotrophic to oligotrophic and productivity gradients. Several flavobacteria groups displayed characteristic habitat distributions, including enrichment of *Polaribacter* and *Tenacibaculum* clades in the relatively nutrient rich western waters, *Leeuwenhoekiella* spp. that were enriched in the more nutrient-deplete central and eastern sites, and the streamlined MS024-2A group found to be abundant across all sites. During the 2015/16 El Niño event, both environmental conditions and microbial community composition were substantially altered, primarily on the western side of the archipelago due to the reduction of upwelling from the Equatorial Undercurrent. When the upwelling resumed, concentrations of inorganic nutrients and DOC at the western surface sites were more typical of mesopelagic depths. Correspondingly, *Synechococcus* abundances decreased by an order of magnitude, while groups associated with deeper water masses were enriched, including streamlined roseobacters HTCC2255 and HIMB11, *Thioglobacaceae*, methylotrophs (*Methylophilaceae*), archaea (*Nitrosopumilaceae*), and distinct subpopulations of *Pelagibaceriales* (SAR11 clade). These results provide a quantitative framework to connect community-wide microbial volumetric abundances to their environmental drivers, and thus incorporation into biogeochemical and ecological models.

## Introduction

Microbes mediate the flux of energy and materials through the ocean (Moran, 2015), yet how environmental conditions structure marine microbial communities, and therefore the suite of biogeochemical and ecological activities they carry out, is only partially understood (Fuhrman et al., 2015; Mende et al., 2017). The Galápagos Islands have long served as a natural laboratory to examine how environmental variation shapes community and population structure. Darwin’s observations of differences in the Galápagos terrestrial habitats and their associated fauna were critical to development of the theory of speciation through natural selection. In contrast to these terrestrial ecosystems, identifying the factors structuring marine microbial communities is less obvious given marine environments are well mixed with low barriers to nutrient and organism exchange. Yet the Galápagos Archipelago is located in a unique oceanographic setting in which several major oceanographic features intersect to create diverse marine habitats with gradients in temperature, inorganic dissolved nutrients, primary production, organic matter composition and concentration, and plankton groups in a relatively close proximity (Jimenez, 1981; Liu et al., 2014; Campoverde et al., 2018) that might make it well suited for identifying marine microbial niche diversification.

**FIGURE 1:**
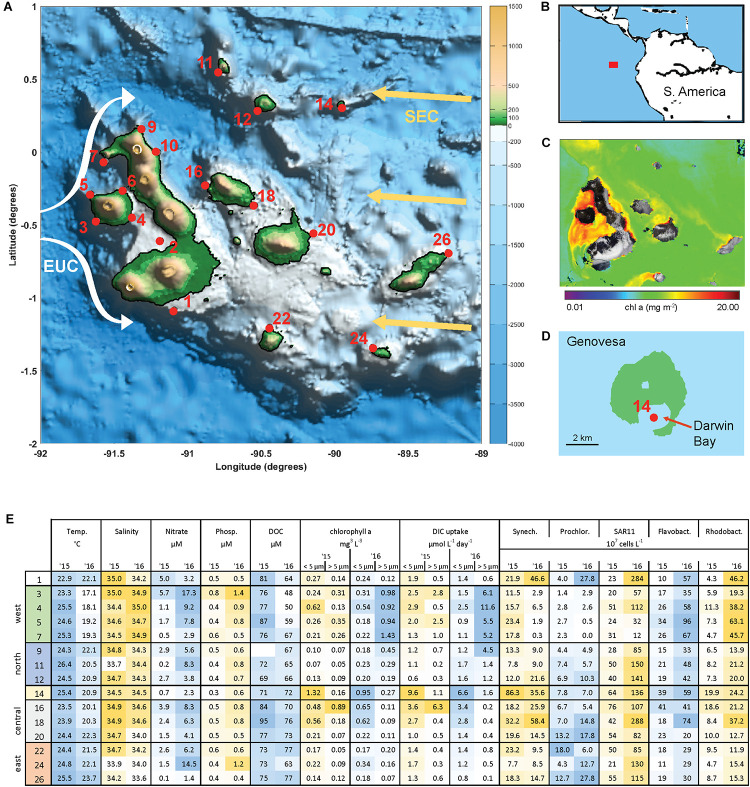
The Galápagos Archipelago lies at the intersection of several ocean currents which create a diversity of marine habitats around the islands. **(A)** Map of the Galápagos platform with sampling locations marked in red and probable current flows of the South Equatorial Current (SEC, yellow arrows) and Equatorial Under Current (EUC, white arrows). **(B)** Location of the Galápagos Archipelago in the Pacific Ocean. **(C)** Chlorophyll *a* concentration from satellite observations (Aqua/MODIS). **(D)** Genovesa Island and sampling Site 14 within the partially collapsed caldera that is now Darwin bay. **(E)** Mixed layer temperate, salinity, inorganic nutrient, size-fractionated chl *a*, and primary productivity (determined by DIC uptake) data, and microbial abundances at sampled stations in 2015 and 2016. Sample site IDs are shown on the left, corresponding with IDs in **(A)** and divided into four main geographical regions. Microbial abundances are based on single-copy gene (*recA*) recovery from the metagenomes normalized to internal standard recovery.

The archipelago consists of 18 major islands lying on the equator approximately 900 km from Ecuador (Figures 1A,B). Current flows across the islands are complex and include influences from the South Equatorial Current (SEC), the North Equatorial Countercurrent, and Peru current (Schaeffer et al., 2008; Liu et al., 2014). The Equatorial Undercurrent (EUC) flows eastward along the equator and directly intercepts the Galápagos platform, generating upwelling of nutrient rich water on the western side of the archipelago. As a result, the archipelago has substantially higher rates of primary production than the surrounding oligotrophic waters of the East Equatorial Pacific (Figure 1C).

These oceanographic features, together with a variety of geological features (Karl et al., 1980; Campoverde et al., 2018), are ripe for creating diverse niches that have unique microbial community compositions and functions. In a study of three Western Galápagos sites in 2014, Campoverde et al. (2018) found pronounced differences in water column properties, organic carbon pools, and microbial community composition, suggesting that there is high spatial diversity in these factors across the archipelago. Further, the islands can experience major shifts in these variables due to seasonal shifts in currents and ENSO events. During El Niño periods, warm equatorial surface water migrates east toward the islands and the EUC upwelling that supports the islands high productivity is reduced, resulting in substantial perturbation of the archipelago ecosystem (Schaeffer et al., 2008).

To understand how patterns in physical, chemical, and biological characteristics shape microbial community composition, we conducted a quantitative metagenomic survey of the Galápagos Archipelago microbiome. We specifically examined, (1) what microbes comprise the community, (2) how the absolute abundances of those microbes are shaped by the diverse physical and chemical setting of the archipelago, and (3) how temporal variability in physical-microbial coupling alters microbial composition. Surface samples were collected across the archipelago over a 2-year period that included the 2015/16 El Niño event. Our results indicate the strong environmental gradients across the archipelago lead to distinct microbial communities, that certain taxa can be used as indicators of water mass history and characteristics, and these communities undergo substantial shifts during El Niño events.

## Materials and Methods

### Water Collection

Samples were collected on board the *M/V* Sierra Negra from October 10th to 24th, 2015 and October 19th to November 11th, 2016 at sites spanning the Galápagos Archipelago and encompassing the diversity of oceanographic features (Figure 1A). Temperature and salinity were obtained by CTD (SeaBird SBE 19plus) profiles and the surface mixed layer was defined by a potential density change >0.35 kg/m^3^ from the surface value (de Boyer Montégut et al., 2004). Reported mixed layer temperature and salinity are averaged values above this depth. Photosynthetic active radiation (PAR) profiles were obtained from a Seabird PAR sensor mounted to the CTD.

A subset of the sites was selected based on their potential to capture the diversity of conditions around the archipelago, and sampled for nutrients, productivity, and metagenomic analysis (chosen sites shown in Figure 1E). Water column samples targeting the surface 50% irradiance level (determined as the 50% proportion of PAR at the surface) were obtained using Niskin bottles (sampling depth range 5–10 m). Cell samples for DNA analysis were obtained by peristaltic pumping one to two liters of seawater through 3 and 0.22 μm filters and freezing at −20°C. A subset of the 3 μm filtrate was collected and preserved in a paraformaldehyde solution (10% final concentration), flash frozen, and enumerated onshore for cell abundances via SYBR staining and epifluorescence microscopy (Supplementary Table 1).

### Primary Production

Triplicate acid-washed polycarbonate bottles (618 ml) were filled with seawater from the 50% light depth, inoculated with isotope, and incubated on deck in tanks for 24 h, beginning between 6:00 and 8:00 am to capture photosynthesis and respiration cycles congruently across sites. The tanks were flushed with surface seawater via a flow-through system and covered with neutral density screening to mimic the incident irradiance depths at which the water samples were collected. Dissolved inorganic carbon (DIC) uptake rates were measured by adding 120 μM ^13^C-labeled HCO_3_ to each bottle prior to incubation (Hama et al., 1983). After incubation, the bottle contents were filtered to capture the plankton community at 24 h of exposure to the trace isotopes. The large size fraction (>5 μm) was filtered onto a 5 μm polycarbonate filter (47 mm) and the remaining filtrate was filtered onto a pre-combusted (450°C for 5 h) GF/F (25 mm) to obtain the small size fraction (≤5 μm). The particles trapped on the 5 μm polycarbonate filters were rinsed with particle-free (0.2 μm filtered) seawater onto a separate pre-combusted GF/F. The filters were dried for 24–48 h in a combustion oven at 60°C, pelletized and stored in a desiccator. Filters were sent to the Stable Isotope Facility at University of California Davis for mass spectrometry analysis. Measurements of particulate carbon (PC) were obtained simultaneously with uptake rates of dissolved inorganic carbon (DIC). Particulate carbon concentrations and ^13^C atom percentages were used to calculate volumetric DIC uptake rates of the different size fractions as according to (Slawyk et al., 1977). Samples were not acidified to remove particulate inorganic carbon.

### Chlorophyll *a*, Inorganic Nutrients, and DOC

Chl *a*, a proxy for phytoplankton biomass, was collected in triplicate by gravity filtering 400 ml of seawater through Isopore 5 μm polycarbonate filters (47 mm) to obtain the large cell size fraction (>5 μm). The filtrate was then filtered onto a Whatman GF/F filter (25 mm) using an in-line vacuum (≤100 mmHg) to obtain the small cell size fraction (≤5 μm). The filters were extracted in 6 ml of 90% acetone and incubated in the dark at −20°C for 24 h. Raw fluorescence values of the chl *a* extracts were measured on a Turner Designs 10-AU fluorometer according to the methods of Brand et al. (1981). Dissolved inorganic nutrients (nitrate + nitrite, phosphate) were measured by filtering 30 ml of water through a 0.2 μm filter, using acid-washed syringes into a polypropylene FalconTM tube. Dissolved nutrient concentrations were analyzed using a OI Analytical Flow Solutions IV auto analyzer by Wetland Biogeochemistry Analytical Services at Louisiana State University.

Dissolved Organic Carbon (DOC) samples were collected at sea from the 0.22 μm filtrate of the cell filtration process. DOC concentrations were measured with a Shimadzu TOC-L_CPH_ analyzer using potassium hydrogen phthalate as a standard for the DOC calibration curve as described in Medeiros et al. (2017a,b). Prior to and alongside sample analysis, both internal blanks and Milli-Q water blanks were run. Analytical accuracy and precision were tested against the Consensus Reference Material (Hansell, 2005) and were better than 5%.

### DNA Processing, Sequencing, and Annotation

Forty-seven samples were chosen for DNA extraction and metagenome sequencing (Supplementary Table 2) with the goal of obtaining even spatial coverage across the archipelago. DNA was extracted from the 0.22 μm filters using a DNeasy PowerWater kit (Qiagen) with modifications. The 0.2 μm filters were removed from the storage tubes using sterilized forceps, placed in the Powerwater bead tube containing lysis buffer, and shaken in a vortex adapter for 5 min. The remainder of the extraction followed the kit manual, and DNA was eluted in 100 μl elution buffer. For quantitative analysis, 4 ng of three genomic standards (*Thermus thermophilus*, *Blautia producta*, *Deinococcus radiodurans*) were individually added to the lysis buffer in 20 μl volumes just prior to starting the extraction, aiming for 1% of total DNA being comprised of genomic standards, assuming c.a. 1,000 ng native DNA in the sample.

Metagenomic libraries were prepared using the KAPA HyperPlus Library Preparation Kit (Kapa Biosystems Scientific, MA, United States) with a 29-min fragmentation at 37°C producing an average fragment size of 395 bp (including ligated adapters). Samples were barcoded with 1 μM NimbleGen SeqCap Adapters (Roche NimbleGen Inc., WI, United States). A dual-SPRI bead size selection selecting for 400 bp fragments was performed after the post-ligation cleanup. Five cycles were used for the library amplification reaction. Barcoded libraries were pooled and sequenced using HiSeq 4000 platform (Illumina, CA). 359 and 364 million 150 bp paired-end reads were generated from the 2015 and 2016 samples, respectively. Reads were processed using the Galaxy bioinformatics platform (Afgan et al., 2016), including quality control with FastQC and trimming low quality read ends with Trimmomatic (sliding window size 10, average quality threshold 20, reads < 50 bp in length were discarded). Paired-ends were assembled using Pear (minimum overlap size 10). Assembled paired-end reads and unpaired forward reads were concatenated into a single file for annotation.

Reads originating from the internal genomic standards were identified via a BLASTn search against the three internal standard genomes (cuttoff: *e*-value < 0.001, %ID > 95%, alignment length > 50% of the read length, bit score > 50) (IMG accession numbers *T. thermophilus* [637000322], *D. radiodurans* [2556921628], *B. producta* [2515154176]). To count the number of internal standard protein encoding genes sequenced, the identified internal standard reads were then annotated via a BLASTx (*e*-value < 0.001) homology search against a database of the internal standard protein sequences, and hits with bit scores < 40 or %ID < 95 were removed. The number of protein-encoding internal standard reads in the sequence library was acquired by counting the remaining hits. Internal standard reads were then removed from the downstream annotation steps.

All non-internal standard reads were taxonomically and functionally annotated using a homology search against NCBI’s Reference Sequence database (RefSeq v84) containing bacterial and archaeal protein sequences via the Diamond search algorithm (version v0.8.18.80; blastx z–salltitles –max-target-seqs 1 –block-size 70 –index-chunks 1) (Buchfink et al., 2015). Duplicate hits and any hits with bit score < 50 were removed with custom scripts. Initially, there was an abundance of reads assigned to a few non-marine organisms such as *Clostridium*, *Escherichia*, and *Salmonella*, and upon closer inspection these reads looked to be of viral origin (functional annotated as ribonucleotide reductases, portal proteins, endonucleases, etc.). We therefore did a homology search of all metagenomic reads against NCBI’s RefSeq viral database (v85) using Diamond to ensure that that no sequences of viral origin were erroneously assigned to bacterial taxa. Bacterial annotated reads were replaced by a viral annotation if the viral hit had a higher bit score.

### Gene Abundances

Recovery of internal standards in the sequence libraries reflects sequencing coverage and was used to estimate gene volumetric abundances for each sample using calculations derived from Satinsky et al. (2013):

$$\left(1\right)\,S_{\text{r}}=\frac{S_{\text{S}}}{S_{\text{P}}}\;\left(2\right)\,R=\frac{S_{\text{r}}}{S_{\text{a}}}\;\left(3\right)\,G_{\text{a}}=\frac{G_{\text{s}}}{R}$$

(1) *S*_r_: Copies of internal standard genome recovered in sequence library.
  *S*_S_: protein encoding internal standard reads in the sequence library.
  *S*_p:_ protein encoding genes in the internal standard reference genome.
(2) *R*: Recovery ratio. The proportion of standard molecules added that were sequenced.
  *S*_a_: molecules of internal standard genome added to the sample.
(3) *G*_a_: Molecules in the sample of any gene category.
  *G*_s_: total reads of any gene category in the sample sequence library.

The volumetric abundance (genes L^–1^) of a gene category (Ga) is determined by dividing by the volume of seawater filtered. The total protein encoding genes in a sample is determined by summing the total number of RefSeq annotated reads and dividing by R.

### Genome Equivalent Abundances

Taxon genome equivalents per liter were calculated by dividing the number of recombinase A genes (*recA;* a conserved single-copy gene) annotated for that taxon by the recovery ratio R then dividing by the seawater volume filtered (Landa et al., 2019). To identify *recA* genes in the metagenome samples, a bacterial RecA protein database was assembled containing proteins from the RefSeq protein database (v.84) annotated with the key words “recombinase RecA,” “protein RecA,” “recombinase A,” or “RecA protein.” Metagenome reads were then compared to the custom RecA database using a DIAMOND homology search (blastx z–salltitles –max-target-seqs 1), with top hits having a bit score > 50 counted as a *recA* gene, and the results checked against the original all RefSeq protein search to confirm the RecA annotation.

### Principal Components Analysis (PCA)

Volumetric abundances of bacterial families were calculated by summing abundances of all *recA* hits within a family. The top 100 families, encompassing 90% of all identified *recA* hits, were then included in the PCA analysis. Community composition was examined by finding the proportion a family made of total genome equivalents in a sample. Zero values were changed to 0.001 and percentages were log_10_ transformed. The PCA was run using the *prcomp* function in R with ‘variables center’ set to true (means centered on zero), and ‘scale’ set to true (standard deviation normalized to 1, all variables have an equal effect on ordination). Community composition differences between samples were tested with a PERMANOVA, using the ‘adonis’ function in the R vegan package (v.2.5.6) with a square root transformation and Bray–Curtis distance calculation. Dispersion was checked using the ‘betadisper’ and ‘permutest’ function to ensure there was no significant difference (*p* > 0.05) in dispersion between the groups being compared.

## Results and Discussion

### Environmental Variability Across the Archipelago

Surface waters (5–10 m) spanning the Galápagos Archipelago were sampled in October 2015 and 2016 (Figure 1A), encompassing a range of environmental conditions (Figure 1E). Western stations had the coldest and most nutrient rich surface waters, owing to the upwelling of the Equatorial Undercurrent (EUC) as it collides with the archipelago platform (Liu et al., 2014). Correspondingly, the western stations had high primary productivity and phytoplankton standing stocks (Figure 1E). By contrast, the eastern stations are relatively isolated from EUC upwelling and are more oligotrophic. Central archipelago sampling stations most often represented a middle state, although several Sites (14, 16, and 18) were notable for their high primary productivity and phytoplankton standing stocks (Figure 1E). Based on our hydrographic and nutrient data and the major oceanographic currents in the system, we divided the archipelago into five regions: west, north, central, caldera, and east (Figure 1E).

Between the 2 years sampled there was substantial variability in hydrographic conditions and microbial composition (Figure 1E). The year of 2015 was classified as an El Niño year based on NOAA’s El Niño 1/2 index of sea surface temperature anomalies in the region that encompasses our Galápagos sites (Reynolds et al., 2002; Santoso et al., 2017). Correspondingly, water temperatures across our sites were on average 2–4°C warmer during the 2015 El Niño than in 2016 (Figure 1E), with some western stations warmed by more than 7°C. Mixed layer inorganic nutrient concentrations were reduced during the 2015 El Niño, with western EUC influenced sites in particular having a 75% NO_3_ and 30% PO_4_ reduction (Figure 1E). As the system returned to a neutral, cooler state in 2016, the western site temperatures decreased and had higher salinities, both indications that EUC upwelling had returned to delivering nutrient-rich midwater to the surface (Figure 1E).

**FIGURE 2:**
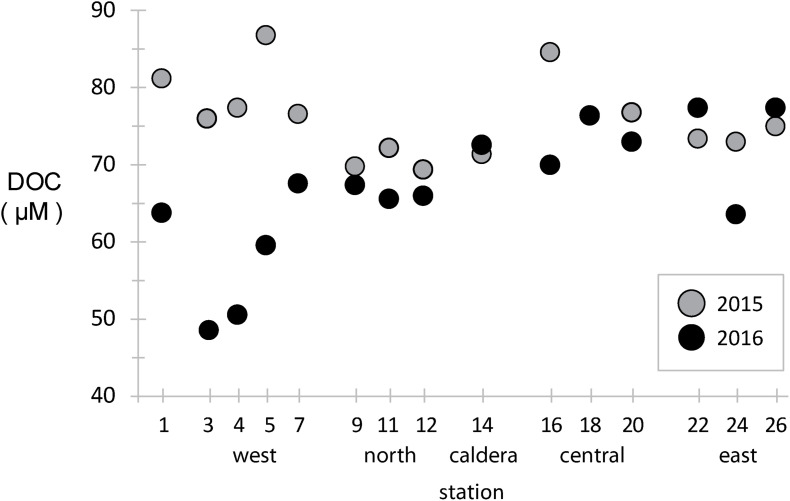
Dissolved organic carbon (DOC) concentrations at stations across the archipelago collected in 2015 (gray) and 2016 (black).

Dissolved organic carbon concentrations were measured across the archipelago in both years (Figure 2). During the 2015 El Niño, western EUC-influenced site DOC concentrations were ∼80 μM, slightly above the average of the rest archipelago, and DOC concentrations were reduced eastward and at a minimum in the north. In 2016, after the system returned to a more neutral state, the DOC paradigm was altered. DOC concentrations in the west decreased to 40–60 μM, a third less than 2015 at the same locations, while mid-island and eastern sites stayed approximately the same.

In general, vertical ocean DOC profiles are highly reproducible across basins and time, with surface DOC concentrations approaching 70–80 μM and decreasing rapidly to ∼40 μM in the mesopelagic (Hansell et al., 2009; Medeiros et al., 2015). Our results in the Galápagos show much of the archipelago has typical surface-like DOC concentrations, except at the western stations where deeper water is likely being upwelled so quickly that surface primary production has yet to increase DOC standing stocks. During an El Niño year, such as observed during our 2015 sampling, reduced EUC upwelling resulted in western stations having a DOC profile more typical of open ocean surface waters.

Together, the hydrographic, nutrient, and DOC data indicate that (1) there is substantial spatial variability in environmental conditions across the archipelago, and (2) El Niño had an extensive environmental effect on many of these sites, most prominently a reduction in the upwelling of nutrient rich waters at the western stations that is the main driver of primary production in this region. Below we explore how the microbial communities inhabiting these waters reflect both the spatial heterogeneity in environmental conditions and temporal dynamics likely driven by the El Niño.

### The Galápagos Marine Microbiome

Stations across the archipelago were selected for metagenomic analysis of the free-living bacterioplankton community (0.22–3 μm size fraction; Supplementary Table 2). A total of 47 metagenomes were sequenced, with 23 metagenomes sequenced in 2015 (with replicates for Sites 1, 2, and 16) and 24 metagenomes in 2016 (replicated sites: 1, 4, 7, 11, 14, 16, 18, 24, and 26; see Supplementary Table 2 for complete details on read quantities and sample information).

**FIGURE 3:**
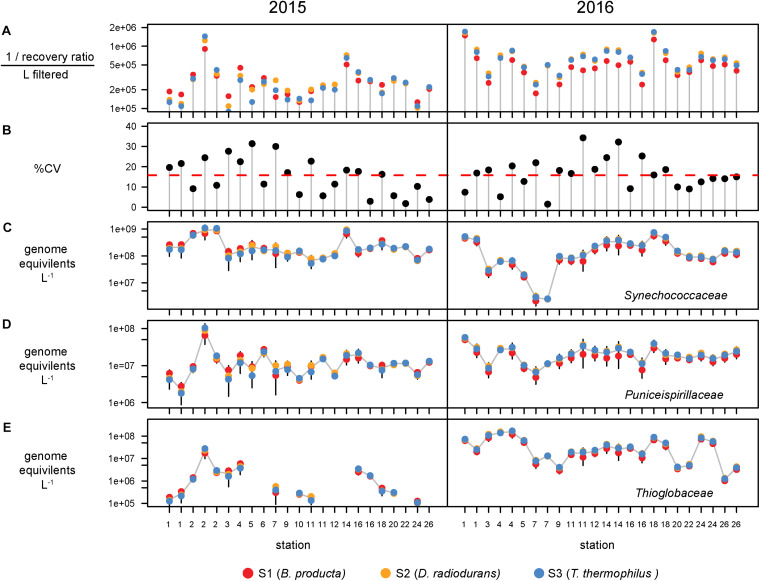
Recovery and variation of the three internal standards and the effect on taxa volumetric abundances. **(A)** Recovery ratio derived from *Blautia producta* (red), *Deinococcus radiodurans*, (gold), and *Thermus thermophilus* (blue) internal genomic standards for the 47 metagenomes. **(B)** Percent coefficient of variation (%CV = standard deviation/mean × 100) of the three standards. Red dashed line shows the mean %CV of the 47s metagenomes. Volumetric abundances of three families, **(C)** *Synechococcaceae*, **(D)** *Puniceispirillaceae* (SAR116 clade), and **(E)** *Thioglobaceae* derived from each internal standard. Error bars denote the 95% confidence intervals (t distribution) determined from the three internal standards for each metagenome.

**FIGURE 4:**
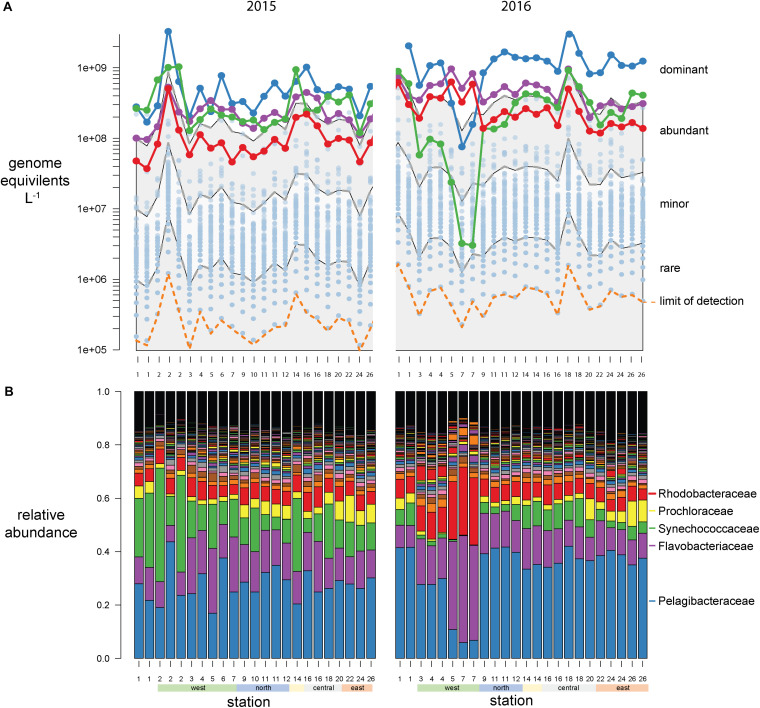
Galápagos marine microbiome composition across the archipelago based on *recA* recovery and binned at the family level. **(A)** Volumetric abundances of all bacterial families. The top 5 families are colored as follows *Pelagibacteracea*, blue; *Flavobacteriaceae*, purple; cyanobacteria, green; *Rhodobacteraceae*, red. The remaining 477 families are plotted as semitransparent blue circles. The distribution of families was categorized into four groups based on their volumetric abundances: dominant, abundant, minor, and rare, and these bins are outlined by the gray and white shading. **(B)** Relative abundances (percent of total site *recA* sequences) of all bacterial families across the sampling stations.

Bacterial abundances were estimated using single-copy *recA* read counts normalized to internal standard genome recoveries (Supplementary Table 2). Individually, the three genomic standards were recovered at similar ratios within a sample (Figure 3A), having an average coefficient of variation of 15% (Figure 3B). Volumetric abundances were calculated separately for each of three internal standards for three bacterial families that are representative of abundant, minor, and rare groups and exhibited a range of abundances across the archipelago are shown in Figures 3C–E. These abundances demonstrate that variation arising from the different internal standards is minimal compared to variation between samples. Summed *recA*-based abundances of all bacterial and archaeal taxa produced estimates of 1.5 and 3.1 billion cells L^–1^ in 2015 and 2016, respectively; corresponding well with direct cell counts (1.6 and 2.2 × 10^9^ cells L^–1^ in 2015 and 2016, respectively; Supplementary Table 1) and were typical of surface pelagic bacterial abundances. We then quantified bacterial community composition by binning genome equivalents at the family level (Supplementary Table 3) and placing them into four categories: dominant (>10^8^ cells L^–1^), abundant (>10^7^ cells L^–1^), minor (>10^6^ cells L^–1^), and rare (<10^6^ cells L^–1^; Figure 4A).

At this broad taxonomic level, the Galápagos microbiome has a typical surface-ocean community composition (Sunagawa et al., 2015; Mende et al., 2017). Two families were consistently in the dominant bin: *Pelagibacteraceae* (SAR11 clade) and *Flavobacteriaceae* (Figure 4). The abundant bin consisted primarily of *Synechococcaceae*, *Rhodobacteraceae*, *Prochloraceae*, and to some extent the *Halieaceae*, *Rhodospirillaceae*, *Puniceispirillaceae* (SAR116 clade), and *Cellvibrionaceae*. These top 9 families in the dominant and abundant bins contained on average 71% of total cell abundances. The minor bin contained a quarter of the 482 families identified, including diverse taxa that are consistently found at each station across both years. The rare bin contained the greatest number of families (424), but their cumulative abundances averaged <0.1% of total genome equivalents and they were often found only sporadically across time and space. The spotty detection of rare organisms is due in part to the limit of detection, which is directly related to sequence depth. In previous quantitative omics studies, the detection limit ranged from 10^5^ to 10^7^ genes or transcripts L^–1^ (Gifford et al., 2011; Satinsky et al., 2014; Wilson et al., 2017), and 10^3^ to 10^5^ rRNA copies L^–1^ in quantitative amplicon studies [internal standard normalized (Wang et al., 2018; Lin et al., 2019; Tang et al., 2019); flow cytometry normalized (Wang et al., 2019)]. In this study, an average abundance of >480,000 cells L^–1^ was needed to detect a *recA* representative in our sequence libraries (Figures 3A, 4A orange dotted line).

#### Variation in Community Composition Across the Archipelago and With Time

**FIGURE 5:**
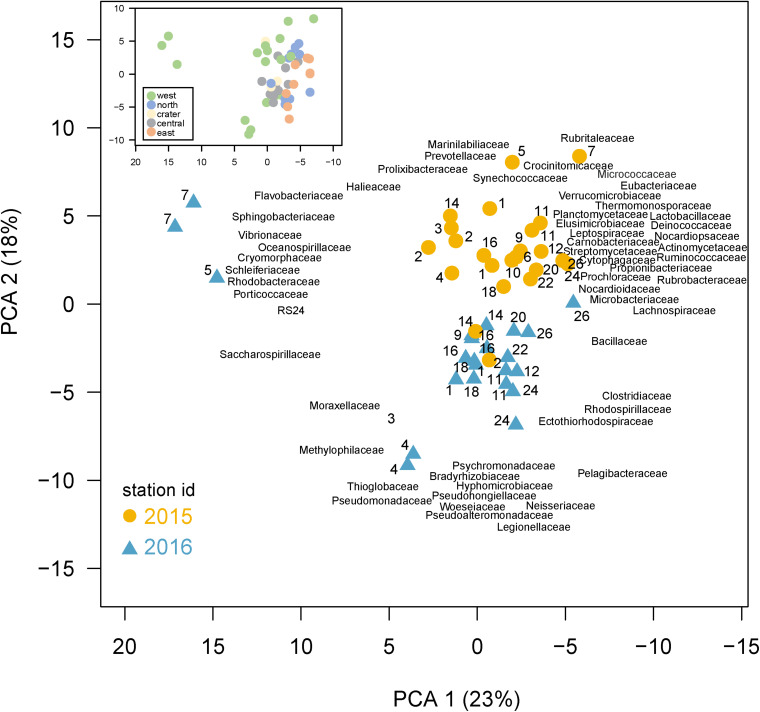
Differences in community composition across the Galápagos Archipelago. Principal components analysis (PCA) based on the *recA* abundances of the top 100 families in the metagenomes. Percent variance explained by each axis is shown in parentheses. Samples are colored based on year collected (2015, gold circle; 2016, blue triangle) with the site number next to the sample symbol. The bacterial family loadings are plotted with left justification. Inset: the same PCA as shown in the main plot, but with samples color-coded according to the geographic region of their collection in the archipelago.

A Principle Component Analysis (PCA) shows bacterial community composition substantially differed across the sites and years, correlating with several environmental conditions (Figure 5). The first two PCA axes explained 41% of total variance and were strongly correlated with site location, environmental parameters, and sampling year. Axis 1 (PC1) reflected the west to east productivity gradient, with the most negative PC1 scores for eastern stations, correlating positively with temperature and negatively with salinity. PC axis 2 shows the samples clearly divided between those collected in 2015 and 2016. A PERMANOVA analysis confirmed community composition differed significantly (*p* < 0.001) between the 2 years, revealing a strong temporal shift in microbial community composition likely due in part to El Niño conditions. The western sites were substantially separated from other sites in the PC analysis and western community composition was significantly different than those in other regions by the PERMANOVA analysis (*p* < 0.05). The PCA analysis thus showed (1) substantial variations in community composition, (2) several taxonomic indicators of a site’s location within the Archipelago, and (3) a substantial response to El Niño served as major shift in composition between these 2 years. Below, we explore specific families that served as taxonomic indicators of a site and how their abundances changed during the 2015 El Niño.

#### Cyanobacteria

**FIGURE 6:**
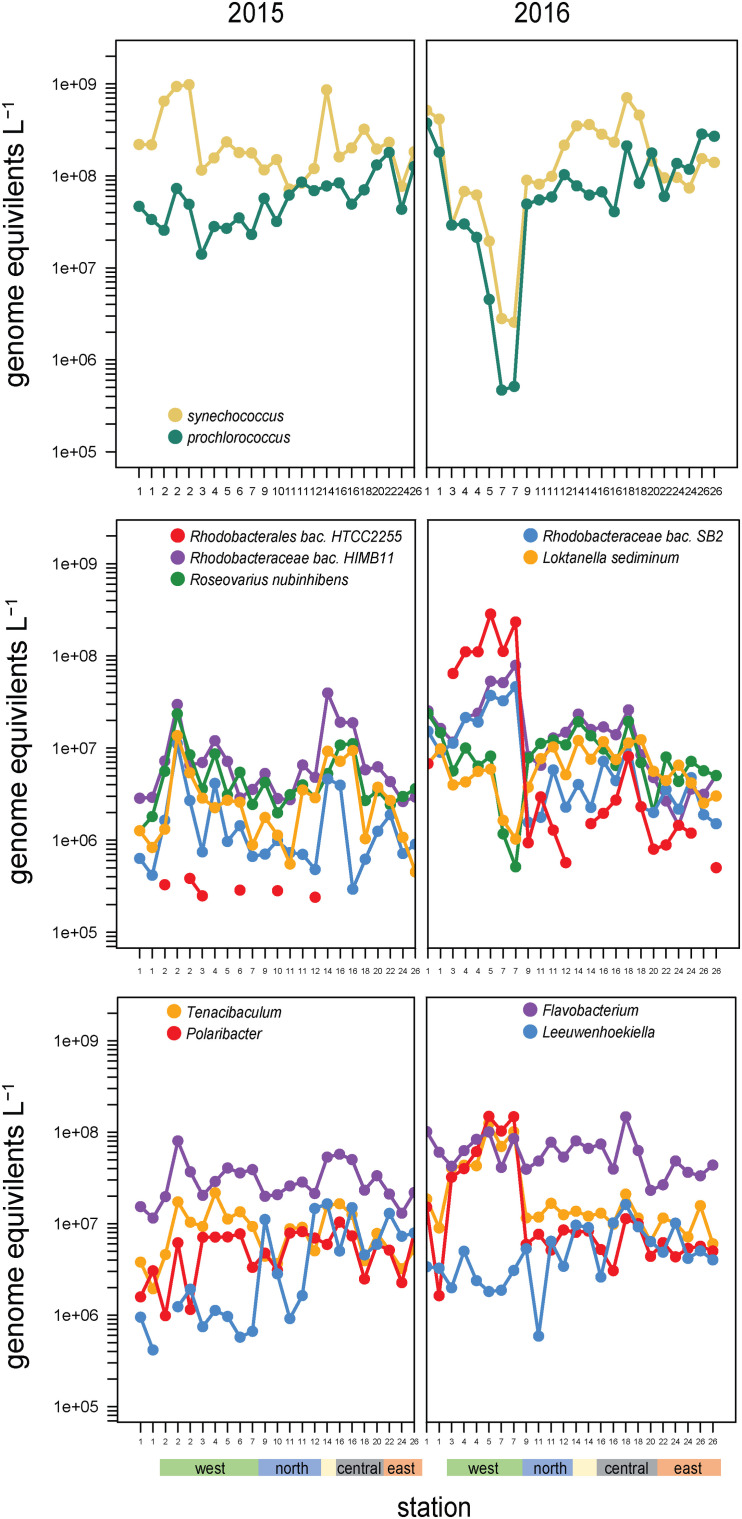
Abundances and composition across the archipelago of select taxa within **(top)** the cyanobacteria, **(middle)** the Roseobacter clade, and **(bottom)** Flavobacteriaceae genera in 2015 **(left)** and 2016 **(right)**. The color bar at the bottom of the graph denotes a station’s geographic region as shown in Figure 1E.

*Synechococcus* and *Prochlorococcus* made up the majority of cyanobacteria (97% of cyanobacteria genome equivalents) and have a distinct spatial distribution across the archipelago, going from a *Synechococcus* dominated system in the west to an even mix of *Prochlorococcus* and *Synechococcus* in the southeast (Figure 6). Notably, this relative evening results from *Prochlorococcus* absolute abundances increasing eastward, while *Synechococcus* cell densities remain consistently high. These cyanobacterial populations are likely major contributors to primary production in the archipelago, given that the <5 μm size-fraction constituted a large fraction of total primary production, especially in the non-western stations and during El Niño (Figure 1E).

High abundances of *Synechococcus* were detected at two separate sites in 2015. Site 14 is located on Genovesa Island within Darwin Bay, a partially collapsed caldera with a sill that restricts interactions with the surrounding ocean (Figure 1D). *Synechococcus* reached 9 × 10^8^ cells L^–1^ in the caldera, becoming the most abundant bacterioplankton in the community. Supporting these molecular observations, high chlorophyll *a* concentrations were measured here, the majority of which was in the small size fraction (<5 μm; Figure 1E). While *Synechococcus* abundances in Darwin Bay were slightly reduced in 2016 compared to 2015 (4 × 10^8^ cells L^–1^), the site still had some of the highest *Synechococcus* densities measured in 2016 (Figure 6).

The western sites experienced order of magnitude shifts in *Synechococcus* abundances between the 2 years. In 2015, *Synechococcus* were dominant community members in the west, second in abundance only to SAR11 clade members. The highest 2015 *Synechococcus* abundances were detected at Site 2 (10^9^ cells L^–1^; Figure 6). In 2016, when EUC upwelling was restored, cyanobacteria densities decreased substantially at western stations, with *Synechococcus* decreasing to 0.3 × 10^7^ cells L^–1^ and *Prochlorococcus* to 0.05 × 10^7^ cells L^–1^ at Site 7, a three order of magnitude drop relative to Site 1 and the largest abundance change between years observed in our dataset (Figure 6).

#### SAR11

**FIGURE 7:**
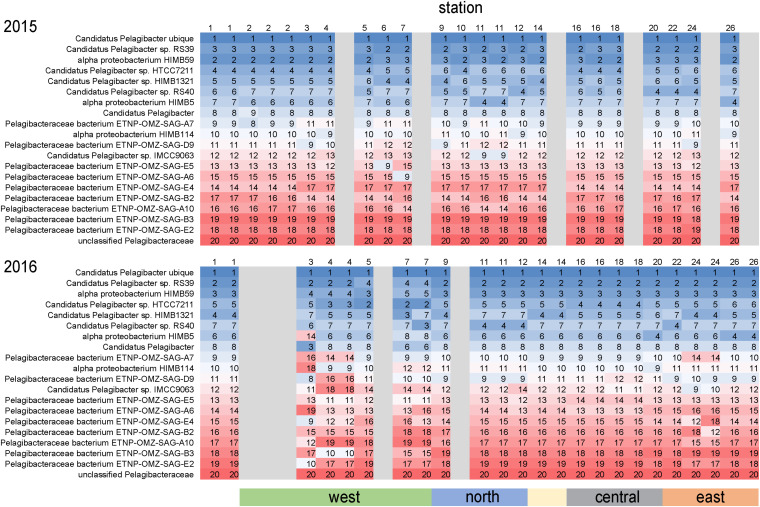
Differential metagenome read recruitment to SAR11 clade reference bins across the archipelago. For each sampling station, the rank order of 20 SAR11 clade reference bins available in NCBI RefSeq v84 is shown in each column and colored from top recruiting (blue) to lowest recruiting (red). Grayed columns indicate no metagenomes were acquired at the site that year.

As is common for marine systems, SAR11 clade members were dominant components of the archipelago community (Giovannoni, 2017). *Pelagibacteraceae* was consistently the most abundant family, averaging 14% of genome equivalents and average densities of 2–5 × 10^8^ cells L^–1^ (Figure 4). The only instance *Pelagibacteraceae* was not a dominant community member was in 2016 at western Sites 5 and 7 during normal upwelling conditions. While SAR11 was consistently abundant across the sites, we detected distinct populations across the archipelago. We examined the distribution of metagenomic read recruitment to the 20 SAR11 reference bins available in the RefSeq database (Figure 7). The *Pelagibacter ubique* bin was always the dominate reference bin, recruiting an average of 70% of all *Pelagibacteraceae* metagenome reads, followed by strains RS39 and HIMB59 making up *ca.* 6% each, and then by a series of SAR11 genomes whose proportion of read recruitment is highly stable across the archipelago. However, in 2016, at the western upwelling stations (Sites 3, 4, 5, 7) and Site 24, there is a distinct shift in the rank order recruitment driven by increased recruitment of several single cell amplified genomes originally obtained from oxygen minimum zones (Tsementzi et al., 2016). This suggests that at actively upwelling sites, the SAR11 community contains unique populations harboring functional genetic potential for dealing with relatively low oxygen and deep-water nutrient conditions.

#### Roseobacters

Members of the *Rhodobacteraceae* family primarily belonging to the *Roseobacter* clade were highly abundant across sites (∼10^8^ cells L^–1^; Figure 4) and binned to diverse taxa (479 reference genomes belonging to >100 genera). Roseobacter abundances substantially increased at EUC upwelling sites in 2016 to become dominant members of the community. These increases were primarily driven by three populations: HTCC2255, HIMB11, and SB2 (Figure 6), which accounted for 40–60% of all *Rhodobacteraceae* genome equivalents in the west. *Rhodobacteraceae* HIMB11 and SB2 are closely related [termed the CHAB-1-5 strains by Billerbeck et al. (2016)], while *Rhodobacteraceae* HTCC2255 lies on the distant evolutionary branches of the roseobacters in the NAC11-7 clade (Suzuki et al., 2004; Newton et al., 2010; Zhang et al., 2016). A comparative genomic study grouped these three strains together with several other Roseobacters prominent in open ocean environments and termed them the Pelagic Roseobacter Cluster [PRC, (Billerbeck et al., 2016)]. CHAB 1–5 strains HIMB11 and SB2 were prominent members of the Roseobacter community across all sites in both years, with typical cell abundances >10^6^ L^–1^. By contrast, HTCC2255 was most often a rare community member. In 2015, HTCC2255 was below our detection limit for 17 out of 23 samples (<3 × 10^5^ L^–1^, Figure 6). However, in 2016, HTCC2255 cell abundances increased from undetectable at Site 1 to >10^8^ cell L^–1^ at EUC upwelling Sites 4, 5, and 7; this was 100-fold higher than at the central archipelago stations it was also detected at in 2016.

In addition to our observations, HTCC2255 is prominent at several sites around the world that have similar steep topographies and upwelling. Phylogenomic analysis by the Genome Taxonomy DataBase (Parks et al., 2018) places HTCC2255 into the *Amylibacter* genus, whose type strain, *Amylibacter marinus*, was isolated from surface waters experiencing strong upwelling with a steep topography off the coast of Muroto, Japan (Teramoto and Nishijima, 2014). HTCC2255 has also consistently been found to be enriched in surface waters of Monterey Bay, CA, United States (Ottesen et al., 2011; Varaljay et al., 2015) that has a similar steep topography and periods of intense upwelling. Together, these findings suggest that HTCC2255 may have a niche for recently upwelled deep waters, potentially thriving on a combination of increased inorganic nutrients and solar radiation availability.

HTCC2255 is notable for its phylogenetic placement at the base of the Roseobacter phylogeny and its streamlined lifestyle (Luo and Moran, 2014). While most roseobacters have large genomes with diverse metabolic and regulatory capabilities, HTCC2255 has a relatively small genome (half the size and number of genes typical of roseobacters), and a relatively restricted set of metabolic capabilities and transcriptional regulators (Newton et al., 2010; Luo and Moran, 2014) suggestive of a more specialist, oligotrophic lifestyle (Giovannoni et al., 2014; Billerbeck et al., 2016). While most Roseobacter genomes lack photo-driven supplemental energy conservation or use aerobic anoxygenic phototrophy, HTCC2255 is one of the only Roseobacter genomes to contain a proteorhodopsin (Newton et al., 2010; Sun et al., 2017).

#### Flavobacteria

The other consistently dominant family in the Galápagos microbiome was the *Flavobacteriaceae*. Like Roseobacters, *Flavobacteriaceae* reads binned to a diversity of genomes (578 reference genomes belonging to >80 genera). Cosmopolitan and abundant flavobacteria included the *Flavobacteria* sp. MS024-2A (notable for its streamlined lifestyle), as well *Nonlabens* and *Arenibacter* genera. EUC upwelling-enriched genera found in 2016 but not 2015, include *Formosa* species and substantial enrichment (>100-fold increase) of *Tenacibaculum* and *Polaribacter* spp. (Figure 6). Notably, while *Tenacibaculum* and *Polaribacter* were consistently found at all sites and times, the specific reference genomes recruiting reads at the EUC sites were hardly detectable at other sites, indicating these particular populations have functional capabilities adapted to deepwater or recently upwelled, nutrient rich environments. Ottesen et al. (2011) also found *Polaribacter* species were substantially enriched in the upwelled waters of Monterey Canyon. Interestingly, we also detected flavobacteria taxa that were distinctly enriched in the eastern stations, most prominently *Leeuwenhoekiella* which was one of the few good heterotrophic indicators of Galápagos oligotrophic habitats (Figure 6). While enrichment of *Leeuwenhoekiella* sp. was spatially segregated from *Polaribacter* and *Tenacibaculum* in the Galápagos along the east-west mesotropic-oligotrophic gradient, this does not likely reflect a streamlined lifestyle given that *Leeuwenhoekiella*, *Polaribacter*, and *Tenacibaculum* reference genomes are relatively large and have metabolic capabilities typical of a generalist lifestyle; future work is therefore needed to identify the specific genome characteristics that lead to the clear niche differentiation among these flavobacteria.

**FIGURE 8:**
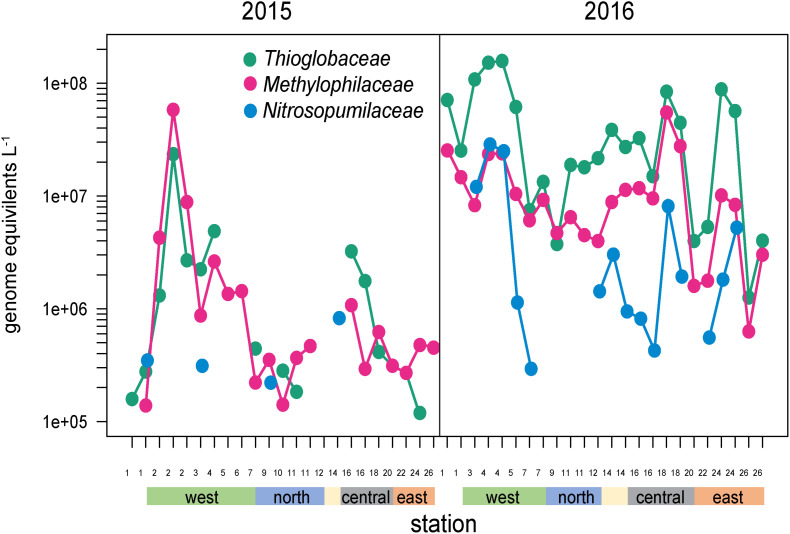
Abundances of *Thioglobaceae*, *Methlophilacea*, and *Nitrosopumilaceae* across the Galápagos Archipelago. The color bar at the bottom of the graph denotes a station’s geographic region as shown in Figure 1E.

Several other groups were substantially enriched at the upwelling sites in 2016 and relatively deplete at oligotrophic eastern sites (Figures 5, 8). Notably, these families could be divided into two groups, one enriched primarily at EUC upwelling Sites 3 and 4 south of Fernandina Island, and the other at EUC Sites 5 and 7 north of Fernandina (Figure 5). *Thioglobaceae* and *Methylophilacea* (Spietz et al., 2019) were a minor component of the community in 2015, but in 2016 increased by greater than 10-fold at sites of strong upwelling (Sites 3, 4, 5) and eastern Sites 18 and 24, while reduced by almost 100-fold again at the far eastern stations. Archaea were relatively rare in our metagenomes, as expected given samples were collected in the upper 5–10 m of the water column and archaea are primarily abundant below the euphotic zone (Karner et al., 2001; Santoro et al., 2019). The most abundant Archaeal family in our data set was the *Nitrosopumilaceae*, which was undetectable in all but four 2015 samples, but become enriched in 2016 at sites 3, 4, 18, and 24 (Figure 8). Taken together, the *Thioglobaceae*, *Methlophilaceae*, and *Nitrosopumilaceae* served as good markers of deep-water intrusion to the surface layer.

### Synthesis

Environmental conditions such as dissolved inorganic nutrients, DOC concentrations, and other hydrographic parameters differed substantially throughout the archipelago, and quantitative metagenomics revealed shifts in the absolute abundances of the microbial communities that correlated with these changes, producing key taxonomic indicators of the archipelago habitats. From these results we can assemble a model of microbial niche diversification across the Galápagos.

During a neutral, non-El Niño year, surface microbial communities on the western edge of the archipelago are strongly influenced by EUC upwelling. Our initial expectation was that nutrient rich upwelled EUC water would drive high primary production and stimulate growth of bacterioplankton typically associated with phytoplankton blooms (Teeling et al., 2012). However, our data suggests intense EUC upwelling rates result in the surface waters immediately bordering the western islands where we sampled to have deep-water characteristics, including high inorganic nutrient and low DOC concentrations typical of the mesopelagic. Correspondingly, the bacterioplankton community at these sites are enriched in deeper water taxa, including archaea and *Thioglobus*, as well as several other Roseobacter and Flavobacteria taxa previously associated with deep water upwelling (Ottesen et al., 2011; Varaljay et al., 2015). Roseobacter HTCC2255 was a particularly strong indicator of deep-water injection, an observation that fits with a potentially distinct niche for this group for rapidly upwelled waters. Even within the cosmopolitan SAR11 clade, western upwelling stations were enriched in deep-water, oxygen minimum zone SAR11 genome-types. Together, this indicates that the western archipelago bacterioplankton communities have not had the surface exposure time needed to develop compositions typically of the upper euphotic zone.

#### Fine-Scale Habitat Diversity

The combination of surface exposure time and complexity of upwelled EUC flow around the archipelago creates fine scale heterogeneity in microbial composition at the western stations. Even western sites in close proximity can have different conditions and community compositions, as exemplified by sites south and north of Fernandina Island. South Fernandina sites (Sites 3, 4) had the coldest temperatures, highest salinities and inorganic nutrient concentrations, and lowest DOC concentrations, and were enriched in deep-water like populations such as *Thioglobus*, archaea, and SAR11 strains. However, north of Fernandina Island (Sites 5, 7), DOC concentrations increased while cyanobacteria and SAR11 populations declined substantially. This area had distinct bacterial communities enriched in roseobacters, flavobacteria, and gammaproteobacteria bacteria, many previously associated with phytoplankton blooms (Teeling et al., 2012). Correspondingly, north of Fernandina Island large cell phytoplankton biomass and DOC concentrations are slightly higher than south, and inorganic nutrient concentrations are drawn down within the mixed layer (Figure 1E). Together these data suggest that the northwestern sites are receiving waters that have been exposed to the surface longer, and thus may have higher abundances of eukaryotic phytoplankton and their associated microbial communities sustained on a more abundant and labile DOC pool.

In a 2014 study at three western archipelago sites in proximity to ours, Campoverde et al. (2018) observed relatively high DOC concentrations (∼92 μM) when temperatures in the west were elevated above typical conditions. This fits with our 2015 observations that warmer western waters had elevated DOC concentrations more typical of surface ocean concentrations. Notably, while our 2015 western sites were significantly warmer than the 2014 observation, the DOC concentrations did not reach the mean 92 μM observed by Campoverde et al. (2018) supporting the authors hypothesis that while temperatures were elevated in 2014, there was still upwelling influence resulting in enhanced phytoplankton production and associated DOC production. Together, both this study and Campoverde et al. (2018) suggest that DOC concentrations are highly heterogenous at the western sites and emphasize the need to better understand the lability of those DOC pools particularly in relation to water mass history.

Further emphasizing the complex fate of EUC upwelled waters is Site 1, just south of Isabela Island, which had different DOC and microbial characteristics than all other western sites, suggesting that EUC flow hadn’t reached this site or is deflected by westerly flowing surface currents. Future work is needed to better understand the fate of EUC waters and their microbial communities as they are advected across the archipelago. What is clear though, is that the environmental conditions and microbial community composition of the western archipelago are substantially altered by El Niño, including warmer waters, a reduction in deep-water nutrient injection, DOC concentrations more typical of the epipelagic, and increased prevalence of taxa typically associated with surface ocean oligotrophic communities.

At the other end of the archipelago, waters surrounding the eastern islands act as oligotrophic endmembers, characterized by lower nutrient concentrations, surface like DOC profiles, and microbial community compositions typical of open ocean ecosystems. Most cells at eastern sites belonged to the SAR11 clade and cyanobacteria. In contrast to the western sites, *Prochlorococcus* is the dominant cyanobacteria in the east, although *Synechococcus* abundance remains high. While roseobacters and flavobacteria were prevalent in the east, they were composed largely of cosmopolitan, oligotrophic ecotypes, such as CHAD-1 and MS024-2A members. Notably, our work showed flavobacteria *Leeuwenhoekiella* seems to occupy a distinctly eastern niche, of interest due to the large genome and copiotrophic lifestyle often associated with this group. The eastern sites were seemingly less affected by El Niño, at least in comparison to the major hydrographic and microbial shifts observed in western sites.

Within the broader archipelago patterns, we observed fine scale habitat diversity, a good example being Darwin Bay located in Genovese Island’s partially collapsed caldera (Site 14; Figure 1D). A ∼10 m deep sill at the mouth of the bay reduces mixing of caldera water with the surrounding ocean, trapping nutrients and microbial biomass, resulting in some of the highest primary production and phytoplankton biomass measurements we observed (Figure 1E). Correspondingly, nitrogen and phosphate concentrations are relatively reduced in the caldera, with moderate DOC concentrations, and *Synechococcus* reaching some of the highest abundances we observed. Interestingly, the caldera did not have a highly distinct bacterioplankton community. High primary production and reduction in horizontal advection by the sill may lead to hypoxic or anoxic conditions in the caldera’s deep water. Future work characterizing the chemistry and biology of Darwin Bay’s deep waters is needed to determine the fate of its organic matter and microbial community.

#### A Potential Island Mass Effect

Several central and eastern archipelago stations were anomalous in their nutrient and microbial characteristics in comparison to nearby sites in the same region (Figure 1E). In 2016, Site 18 located northwest of Santa Cruz Island was enriched in Roseobacters (particularly HTCC2255), *Thioglobus*, and *Methylophilacea*. Similarly, Site 24 located northwest of Española Island, was also enriched in *Thioglobus*, *Methlophilacea*, deep-water SAR11 genome-types, and a notable increase in *Thaumarchaeota*. Site 24’s microbial community clustered more with the northwestern stations in our PCA analysis and had decreased DOC concentrations (63 μM). These taxa and environmental conditions are more characteristic of western sites receiving deep-water injection from EUC upwelling.

Island wake-induced primary production may explain these stations’ anomalies. In the season we sampled, there are consistent winds coming from the southeast, which likely sets up island wakes on the leeward sides of the islands. Islands wakes are known to induce ‘mass effects’ in which eddies on the leeward side enhance mixing and deep-water nutrient injection and increased primary production (Hasegawa et al., 2009; James et al., 2020). Based on our microbial and environmental observations, as well as historical satellite chlorophyll measurements (Figure 1C), we hypothesize that island wakes are contributing to surface nutrient injection and increased microbial activity on the leeward side of several of the Galápagos Islands, such as Española and Santa Cruz (Sites 18, 24). These island wakes may be another important mechanism supporting primary production and structuring microbial communities, and future work is needed to understand the magnitude of their influence in the Galápagos Archipelago.

### Conclusion

Studies of the Galápagos revolutionized our understanding of evolution and ecology by examining biological heterogeneity among terrestrial islands ecosystems. Here we show the marine waters surrounding the islands also have physical and chemical gradients that promote distinct microbial habitats within relatively close proximity. The Galápagos Archipelago thus serves as a natural laboratory for the bottom-up factors that structure marine microbial communities and lead to niche diversification, as well as the potential for certain microbial taxa to be indicators of water mass types. This work also connected volumetric abundances of microbial taxa to environmental niches, working toward a quantitative framework for incorporating microbes into ecosystem and biogeochemical models. Finally, the substantial physical, chemical, and biological shifts the archipelago experienced during El Niño serves as a model for microbial responses to a warming ocean due to climate change.

## Data Availability Statement

The datasets generated for this study can be found in online repositories. The names of the repository/repositories and accession number(s) can be found below: https://www.ncbi.nlm.nih.gov/, PRJNA640218.

## Author Contributions

SG, HS, and AM designed the field sampling and collected field data. SG, BS, and KD processed samples and acquired metagenome datasets. PM processed and analyzed the DOC data. LZ and SG processed and analyzed the metagenome datasets. SG, LZ, BS, PM, HS, and AM wrote the manuscript. All authors contributed to the article and approved the submitted version.

## Conflict of Interest

The authors declare that the research was conducted in the absence of any commercial or financial relationships that could be construed as a potential conflict of interest.
